# No effect of weight cycling on the post-career BMI of weight class elite athletes

**DOI:** 10.1186/1471-2458-13-510

**Published:** 2013-05-27

**Authors:** Laurie-anne Marquet, Morgan Brown, Muriel Tafflet, Hala Nassif, Rémy Mouraby, Samir Bourhaleb, Jean-François Toussaint, François-Denis Desgorces

**Affiliations:** 1 Institut de Recherche bioMédicale et d’Epidémiologie du Sport (IRMES), INSEP, 11, avenue du Tremblay, 75012, Paris, France; 2 UFR STAPS, PRES Sorbonne Paris Cité, Université Paris Descartes, 1 rue Lacretelle, 75015, Paris, France; 3 INSERM, U970, Centre de Recherche Cardiovasculaire de Paris (PARCC), 56 rue Leblanc, 75012, Paris, France; 4 Centre d’Investigation en Médecine du sport (CIMS), Hôtel-Dieu, Assistance Publique-Hôpitaux de Paris (AP-HP), 1, place du Parvis de Notre-Dame, 75004, Paris, France

**Keywords:** Weight cycling, BMI, Post-career, Elite athletes

## Abstract

**Background:**

Repetitions of pre-competition weight-loss diets done by athletes participating in weight class sports can be regarded as periods of weight cycling. The aim of the present study is to identify the long term post-career (22 years) evolutionary profile of athletes’ BMI after such weight cycling.

**Methods:**

One hundred and thirty-six retired French athletes who participated in major international competitions in weight class sports (rowers, wrestlers, boxers, and judokas) were included. Former and current body mass, height, dietary characteristics during the career (annual frequency, amount of weight lost), current physical activity, and answers on the eating-attitude test were collected by phone interview (consistency was tested by comparison with measured weight). We performed ANOVA tests for comparison between groups (sport, dieting), post-hoc tests (Bonferroni test) to identify differences within groups. BMI’s changes were treated using a mixed model.

**Results:**

The recorded weight changes did not depend upon time since retirement. Between 18 y and 50 y, athletes’ BMI increased by 3.2 kg/m^2^ compared to the 4.2 kg/m^2^ increase in the general population. This increase was independent of the number of diets during the career. Retired athletes declared a mean weekly physical activity of 4.8 h ± 4.3. The eating-attitude test showed low scores for all sports without any correlation to diet characteristics.

**Conclusion:**

Weight cycling during an athletic career does not induce a massive weight gain after retirement, probably due to the high level of physical activity still practiced after retirement by these athletes.

## Background

Weight cycling can be considered as periods of weight loss interspersed by periods of subsequent weight gain [1]. The long-term caloric restriction maintaining proper nutrition appear to be beneficial for the lifespan [2], whereas weight cycling may be more deleterious [3,4]. The effects of weight cycling have been principally observed in obese populations with particular focus on post-diet weight maintenance [5,6].

The range of weight regain following diets remains under debate. Some studies show a return to baseline values [7,8] and other report no weight regain [9-11]. Obviously, the major concerns of weight cycling deleterious effects in a public health perspective remain in a possible fat mass increase [3] or in a decrease in resting metabolic rate [12].

Weight control is necessary for elite athletes, especially for those who are required to weigh-in within a given category on a competition day. This weight control occurs before each competition, during every season throughout an athlete’s career, in order to perform in the optimal weight class, or in the strategically chosen weight class, on the day of the event. This behavior can be implicitly assumed as a weight cycling phenomenon. Athletes need to find a perfect balance between weight and performance. Wrestling, boxing and judo are combat sports with seven to eleven weight categories. In rowing the lightweight class is defined for athletes weighing less than 72.5 kg for men and 59 kg for women, whereas heavyweight athletes have no weight constraint. Athletes’ weight loss may be rapid or more gradual depending on the strategies; restrictions of food and fluid intakes are often associated with maintained or enhanced training volume. For combat sports the weigh-in occurs the day before (wrestling, boxing) or the day of the competition (judo: 3 to 6 hours before the competition). This delay between weigh-in and the match may allow acute recovery strategies, subsequent to severe food and fluid intake restrictions with dehydration (during the days just before the weigh-in) associated with vigorous exercises done with the use of sweat suits or saunas [13,14]. In lightweight rowers, the delay between the weigh-in and the start of the first race of the day is shorter (no more than 2 h) [15]. The management of weight loss is then different (less use of dehydration, less weight to lose the day of the event) [16].

Only a few studies have examined the effects of weight cycling in athletic population. During a sport season, female rowers [17] lost weight before the competitive events, but always returned to their previous weight without exceeding it, like wrestlers who showed no long-term effect on weight gain [18]. Nevertheless, some competitive sports that induce a particular pressure on body weight could stimulate eating disorders during and after the career. Some studies suggested that elite athletes may be at greater risk of developing eating disorders (anorexia nervosa, bulimia) especially in aesthetic or in combat sports [19]. Conversely, in a study of 2067 high level French athletes, Schaal et al. [20] noted that only 4.9% of the athletes involved in combat, weight-class sports, met the diagnostic criteria for an eating disorder. A similar observation was made in American college athletes [21]. To date, little is known about the duration of eating disorders after sport retirement [22]. The high amount of physical training undertaken during an elite career is assumed to provide major health benefits notably against chronic diseases [23]. Many studies reported and discussed the risks associated with the cessation of physical activity in former elite athletes but only in the short-term analysis (less than 1 year) [24,25].

The monitoring of weight or health status of former athletes over prolonged periods remains a real challenge and to our knowledge has not been studied in French athletes. Therefore, the aim of this study was to identify the long-term change of the BMI profile in French wrestlers, boxers, judokas and rowers after having experienced frequent pre-competition diets over their career. Furthermore, our aim was to compare the BMI changes to the general population. Body Mass Index (BMI) is normally associated with non-athletic population, but some studies show BMI is likely to be a valid measure for athletes too [26,27]. It can be used to highlight each sport constraint and is the best indicator to compare participants with marked differences in height and weight. We tested the hypothesis that weight cycling did not induce a larger BMI increase in elite athletes compared to the general population after the competitive career with respect to the competitive sport group.

## Methods

### Participants

The present study is a longitudinal research dealing with current and retrospective information from a cohort. The studied population was recruited in 2009 and composed of retired French athletes, who had been selected for international competitive events (Olympic games 1980–2004; world championships 1978–2003; European championships 1978–2003) in sports with competitive weight categories. Athletes were identified based on French federal databases of sport performances and were classified according to the weight category in which they had their best performances. 61 lightweight rowers, 75 heavyweight rowers, 101 wrestlers, 94 boxers and 100 judokas matched the criteria and were eligible to participate in the study. Subsequently, only those with up-to-date contact information were reached to participate in the study. Finally, after affirmative answers our final population regrouped 142 elite athletes (24,1% respondents). 104 of the total sample (73%) (84 men; 20 women) had to reach weight limits to compete: lightweight rowers (26 men, 7 women), wrestlers (28 men, 5 women), boxers (22 men), judokas (8 men, 8 women), were classified as the dieting group. Athletes that did not undertake diets during their career composed the non-dieting group (n = 38, 27%): 31 rowers (heavyweight: 21 men, 8 women and lightweight: 1 man, 1 woman), 4 wrestlers (1 man, 3 women), 1 boxer (1 man) and 2 judokas (2 men).

Written informed consent was received from all subjects after verbal and written explanation of the experimental design and potential risks and benefits of the study. The study was conducted according to the ethic guidelines of the Paris Descartes University and it was declared and approved by the Paris Cochin ethics committee (Paris, France). No direct intervention was conducted on subjects therefore no subjects’ insurance was required by the ethic committee.

### Data collection

Interviews were conducted by phone with the participating retired athletes. We build up our own specific database for the aim of the study including the following collected variables: body mass, height, current weekly physical activity (in hours), characteristics of the diets during the career (annual frequency, amount of weight lost). Body mass and dietary characteristics were asked at current and various time periods of their sport career (at 18 years old, 20-25 y, 26-30 y, 31-35 y, 35-40 y, 41-45 y, 46-50 y and 51-55 y). Total cumulative body weight loss throughout the career was calculated by adding the amount of body mass lost at each diet. To obtain the mean post-career body mass gain, we calculated the difference between the body mass at the age of retirement and the body mass the day of the interview. The mean body mass during career was calculated from the average over each age period. BMI was calculated as body mass (kg) divided by the square of height (m). The BMI delta was calculated as the difference between the BMI at the date of the interview with the BMI at 18 years.

We classified the data according to 3 periods: (1) during the career, (2) at the end of the career and (3) post-career. The period “During career” started at 18 years of age for all the athletes and ended at the last 5-year period before the age of retirement for each athlete. The period “End of career” consisted of only one age class, containing the retirement age. The “Post-career” period was considered from the retirement to the day of the questionnaire.

The participants also completed a questionnaire for assessing their current eating attitude (eating attitude test, EAT-26) [28] in order to identify traits of eating disorders [29]. The test is composed of 26 questions in three subscales (dieting, binging, oral control). A score exceeding 20 represents a positive tendency toward eating disorders.

The reliability of the data provided by the interview was assessed by comparison with direct measurements conducted in the month following the phone-interview in 27 representative participants of the total sample (9 lightweight rowers, 4 heavyweight rowers, 6 wrestlers, 3 boxers, 5 judokas; 22% of them were non-dieting athletes compared with 27% in the original sample). These participants were designated by the researchers. A single trained investigator performed the body composition measurements. The body mass was measured to the nearest 0.1 kg by an electronic scale. To analyze a possible recall bias in reporting past body mass, we examined the intra-class correlations between reported and measured body mass.

For our analysis, the evolution of the BMI of the participants was compared between sports, between dieting category and with the French general population trend, as the subjects were all French athletes. The BMI of the general population was collected from Obépi 2009 [30].

### Statistical analysis

Comparisons of collected data between sports were carried out using ANOVA tests. If a main effect was observed, post-hoc tests (Bonferroni-tests) were applied to identify differences between groups. Data of BMI’s change (for sports, diet, number of diets) at each age period were treated using a mixed model (SAS Proc mixed) with a repeated effect of the age (*ie*. “time”) and using an auto-regression of order 1 variance-covariance (AR (1)) matrix, which takes into account that data are more correlated between two consecutive periods than between two non-consecutive ones. Differences were considered significant for p values ≤ 0.05. Results are expressed as mean (± SD). After checking on non-significant statistical differences and due to the small number of women in our sample, gender has not been separated in the analysis. All analyses were done using SAS and R 2.10.1 softwares.

## Results

### Career, anthropometric measures, diets and physical activity level by sports

The anthropometric variables and other descriptive variables according to the sport are shown in Table 1. Significant differences were found for the height and the mean body mass during the career between sports*:* rowers, especially the “heavyweight”, were taller and heavier than the athletes in fighting sports (p < 0.01). Self-reported body mass agreed closely to measured body mass (intra-class coefficient = 0.99). The career duration differed according to sport (p < 0.01): boxers had a longer career than heavyweight rowers (p < 0.05) and judokas (p < 0.05). Judokas also ended their career at a younger age than light- and heavyweight rowers and boxers (p < 0.05) but with no significant difference with wrestlers (p = 0.12).

**Table 1:** Subjects (dieting – except for the heavyweight rowers) characteristics for anthropometry, diets and physical activity

	**Rowing**		**Wrestling**	**Boxing**	**Judo**
	***Lightweight***	***Heavyweight***	***(n = 33)***	***(n = 22)***	***(n = 16)***
	***(n = 33)***	***(n = 29)***			
Age *(years)*	38.45 ± 7.33	38.6 ± 7.77	41.94 ± 6.57	41.64 ± 4.86	41.06 ± 6.19
Height *(m)*	1.79 ± 0.06 ^$ #‡^	1.87 ± 0.08 ^* # ¤ ‡^	1.71 ± 0.07	1.74 ± 0.06	1.70 ± 0.08
Mean body mass throughout the career (*kg)*	70.59 ± 6.29 ^$ ¤^	82.08 ± 95.85 ^* # ¤ ‡^	70 ± 12.22	62.85 ± 7.88	68.05 ± 10.20
Career length *(years)*	10.09 ± 3.25	9.61 ± 3.76	10.67 ± 4.06	13.35 ± 3.48 ^* $‡^	9.25 ± 2.17
Age of retirement *(years)*	28.09 ± 3.25	27.96 ± 3.86	28.67 ± 4.06	31.35 ± 3.48^* $‡^	27.25 ± 2.17
Mean body mass loss per diet (% body weight)	4.5 ± 2.33	*No data*	7.38 ± 2.79^* ¤ ‡^	5.07 ± 1.82	4.66 ± 1.33
Number of diets during career	4.5 ± 2.33 ^# ¤ ‡^	*No data*	15.54 ± 7.3^¤^	26.36 ± 5.96	24.87 ± 14.01
Total body mass lost in career *(kg)*	23.11 ± 18.96^# ¤ ‡^	*No data*	67.74 ± 52.81	72.27 ± 37.69	59.31 ± 48.66
Physical activity level post-career *(h/week)*	4.86 ± 4.24	*No data*	4.86 ± 3.39	5.41 ± 6.43	3.81 ± 2.14

Values are mean ± SD. *: significant difference with the lightweight rowers group; : significant difference with the heavyweight rowers group; #: significant difference with the wrestlers group; ¤: significant difference with the boxers group; ‡: significant difference with the judokas group.

The body mass lost per diet relative to the body mass differed between sports (p < 0.01). Larger loss appeared in wrestlers (7.4% ± 2.8; p < 0.01) compared to other sportsmen (lightweight rowers: 4.5% ± 2.3, boxers: 5.1% ± 1.8, judokas: 4.7% ± 1.3). The number of diets during the career differed according to the sport group (p < 0.01). Boxers reported undergoing more diets during their career than wrestlers (p < 0.01) and lightweight rowers (p < 0.01). Lightweight rowers cumulated the smallest weight loss over the entire course of their career than the other sports (p < 0.05 compared to judokas, p < 0.01 compared to wrestlers and boxers). There was no statistically significant difference (p = 0.7) when the analysis was only conducted among the fighting sports.

Retired sportsmen declared weekly physical activity as follow: boxers practiced 5.41 h/week (± 6.43); judokas: 3.81 h/week (± 2.14); wrestlers: 4.86 h/week (± 3.39); rowers: 4.86 h/week (± 4.24). We found no statistical difference for the number of hours practiced per week post career between the sport disciplines (p = 0.7).

### Evolution of weight and BMI

**Figure 1:**
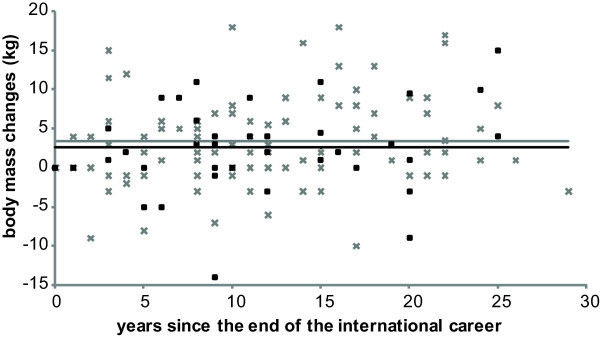
**Body mass changes as a function of years since the end of the career for men and women.** Values presented for dieting (*grey cross*) or non-dieting athletes (*black square*) Mean values for each group are represented by a line: no dieting group (*black line*) dieting group (*grey line*).

Figure 1 presents athletes’ body mass changes from the career end to the interview day according to the time duration of this interval. There was no statistical difference in the mean body mass gain between participants who performed diets during their career and participants with no dieting (p = 0.3). The mean body mass gain (kg) for the dieting group was 3.4 ± 5.6 and 2.5 ± 5.8 for the non-dieting group (p = 0.4). The variations in body mass gain in each group are mixed but stay moderate. 35% of the retired athletes interviewed had a BMI > 25 (48 participants: 16 wrestlers, 7 heavyweight rowers, 6 lightweight rowers, 6 boxers and 6 judokas) and 3% (4 wrestlers) had a BMI > 30.

There was no correlation between body mass change since the career end and the physical activity volume (p = 0.65).

**Figure 2:**
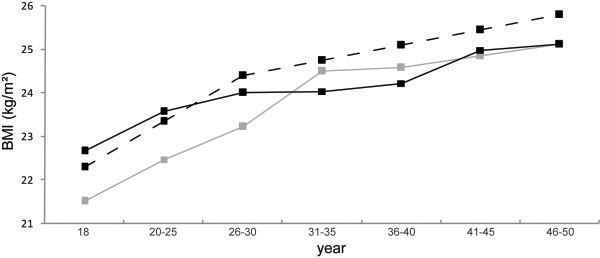
**BMI change with age group for men and women–for dieting sportsmen athletes (*grey line*) who experienced dieting before competition: associated to weight cycling, for no dieting sportsmen athletes (*black line*)who never made diet during their career and for the general population (*black dotted line*).**

The pattern of BMI changes for dieting athletes was similar to non-dieting athletes (p = 0.38 for the interaction diet × time) (Figure 2).

**Figure 3:**
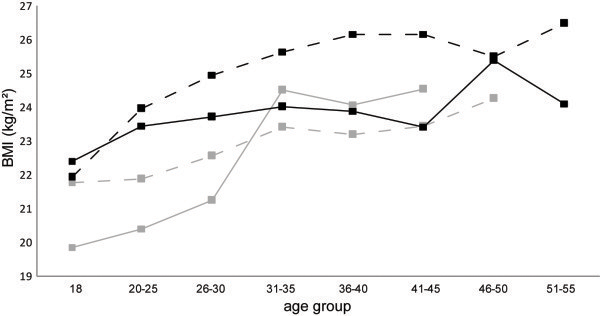
**BMI change as function of age and sport group – for lightweight rowers (*grey dotted line*), wrestlers (*black dotted line*), boxers (*grey line*)and judokas (*black line*), for men and women.**

From the start of their career (18 years) until 2009, the BMI of elite athletes increased by 3.2 kg.m^2^ ± 1.6 (from 21.6 ± 1.2 to 24.8 ± 1.2 kg.m^2^) (lightweight rowers: 2.30 ± 0.87; heavyweight rowers: 2.75 ± 1.8; wrestlers: 4.87 ± 4.2; boxers: 3.78 ± 2.9; judokas: 2.05 ± 1.1) (Figure 3)*.*

The mixed model analysis describes that the BMI change differed between sports (interaction sport × time, p < 0.01 for rowers and judokas in comparison with wrestlers). The period of the career (during career/end of career/post-career) also had a significant effect on the BMI evolution (p < 0.01). BMI during the career was lower than BMI post-career.

The evolution of BMI follows the same trend regardless of the number of diets during the whole career *(Data not shown).* With the mixed model analysis, there is no effect of the number of diets on BMI (p = 0.4).

The difference between the BMI at 18 years old and the BMI at the time of the study is not significant when adjusted according to the sport group (p = 0.58).

### EAT-26 questionnaire

**Figure 4:**
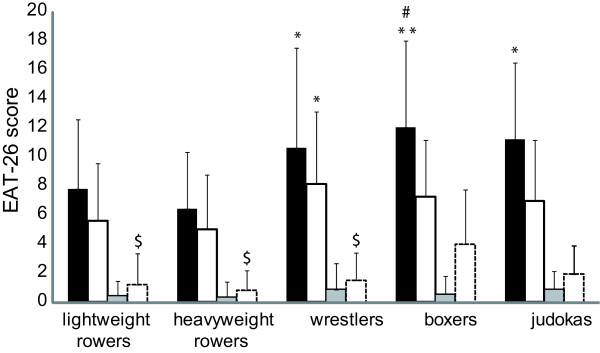
**Scores obtained at the eating attitude test (EAT-26) (*black bar*) and subscales: dieting (*white bar*), bulimia (*grey bar*), and oral control (white open bar ), for men and women.** Mean values of the scores sorted by subscales and sport (+SD). #: significantly different from lightweight rowers for the score obtained at the EAT-26 questionnaire. *: significantly different from heavyweight rowers for the score obtained at the EAT-26 questionnaire and the dieting subscale. $: significantly different from boxers for the oral control subscale.

The mean score obtained on the EAT-26 questionnaire was significantly different according to the sport (p < 0.01). There is a significant difference between sports for the dieting subscale (p < 0.05) and the oral control subscale (p < 0.01) but not for the bulimia or the food preoccupation subscale (p = 0.47). The dieting and the non-dieting groups differed for the EAT-26 score (p < 0.01), the oral control (p < 0.05) and the food preoccupation subscales (p < 0.01) but nor for bulimia (p = 0.07). Total EAT-26 score was higher in boxers (12.1 ± 5.9) than rowers (heavyweight: 6.5 ± 3.9, p = 0.004 and lightweight: 7.8 ± 4.8, p = 0.048) (Figure 4). Heavyweight rowers had lower scores compared to the wrestlers (10.7 ± 6.9, p = 0.031), the boxers (p = 0.004) and the judokas (11.3 ± 5.2, 0.04). Combat sports differed only for the oral control subscale (p < 0.01). No correlation between the questionnaire results and the number of diets or the total weight loss during the career was observed. Only 0.4% of the participants (3 wrestlers, 2 boxers, 1 judoka, all men) had a score higher than 20. All rowers obtained a score below 20.

## Discussion

This study shows that repetitive weight loss and regain during the career of elite athletes does not result in a massive body mass gain after retirement from sport competition. Our data also show high physical activity levels and low eating disorder scores in elite retired athletes. The BMI change after the retirement is influenced by the sport group but not by the number of diets performed during the career and the dieting or non-dieting behavior.

The studied population regrouped 136 French athletes of the main weight-class sports competing in striking (boxing) or grappling (judo and wrestling) combat sports and one endurance sport (rowing) with data corresponding to the sport career and after retirement.

The calculated correlation between the declared and the measured body mass suggests a good reliability of data from interviews. In addition, this result reflects the good ability for former athletes to assess their own body weight probably because they focused on it during their whole sport career.

The hypothesis stating that repeated weight loss may result in a mid-term body mass gain (and consequently a BMI increase) is not supported by our study. The BMI of retired athletes remains under the mean BMI of the general population matched by age group. The body mass gain between the end of the career and the day of the questionnaire was not different between the dieting and the non-dieting groups. Similar results were reported in other studies on former wrestlers in comparison with other athletes [18] and on the short-term effects (6-12-months) of diets in athletes who were not necessarily participating in weight class sports [31].

Our study brings a new approach through a mean follow-up of 22 years (since their age of 18 y and the time of the study) (mean age: 40.4 y ± 6.9). From the age of 18 years to 50 years, athletes gained 3.2 kg/m^2^ compared to 4.2 kg/m^2^ gained by the general population. It follows the BMI change in the population of developed countries with an initial growth until a peak at 55–60 years and a subsequent decline. Participants declared a mean 4.7 h/week of sport practice, which is higher than the 2 h-3 h/week of moderate physical activity recommended for general population [32]. As recently reported, former elite athletes appear to maintain a high volume of physical activity [33] that could explain the limitation in the amount of body mass gain observed after retirement.

Our findings are in conflict with a previous study, reporting that weight gain post career in elite athletes having experienced weight cycling was higher than those observed in non-dieting athletes [34]. In our study, non-dieting athletes presented a higher BMI due to the type of sport they represent (mostly heavyweight rowers), which leads to specific morphological particularities (they are heavier and taller than the other athletes). In this previous study, retired dieting athletes were reported with lower physical activity level than non-dieting athletes whereas such difference did not appear in our data. The control populations also differ between both studies: in the French population, 30% of individuals are overweight [30], in contrast with Saarni [34] who has a control group with 50% of overweight individuals. Moreover, in Saarni et al. participants competed from 1920 to 1965 whereas in the present study participants competed from 1978 to 2003. This could be explained by sport constraints as well as by athletes monitoring that differed between these two periods. Guidelines for nutrition behavior and lifestyle have markedly changed during the 20th century according to society and population changes that could have influenced the athletes weight management at the sport competition retirement [35].

The BMI trend is the same in dieting athletes, non-dieting athletes and in the general population. According to the declaration of athletes, we can stress on the minor amount of body mass lost during each diet. The elite athletes need only to adjust their mass up to a few kilos to enter their weight class. Wrestlers lost a mean of 7% of their body mass per diet, in contrast with the lightweight rowers who make the lightest diets with a mean loss of 4.5% of body mass probably due to the time of the weigh-in (2 h before competition). They can’t support a massive dehydration. Our results are in accordance with a study which reported a mean loss of weight of 6.9% for wrestlers [36]. The BMI change statistically differs between the disciplines (despite similarities) and might depend on the sport-specific training developed by the athletes. In our population sample, wrestlers have the highest BMI that could be associated to their previously reported higher trunk strength when compared to judokas [37]. Conversely, recall of body mass during the career showed that boxers had the lowest body mass and BMI as they principally belong to lightweight categories. Boxers show a higher body mass increase at the end of their career, without exceeding the values of the general population. Heavyweight rowers presented a high BMI, being taller (mean 1.91 m ± 0.05) and heavier (86.97 kg ± 5.65). According to the competition level, rowers anthropometrics may be linked to performance [38,39].

Athletes competing in aesthetic or in combat sports have been assumed to be at greater risk for developing eating disorders that could influence BMI change post career [19]. In this present study, no correlation is found between the score obtained at the EAT-26 questionnaire, the number of diets and the total body mass lost during the career. However, dieting athletes showed the higher scores in the subscale related to the food control. As observed in a larger population of athletes currently engaged in competition [20], our study does not show high levels of eating disorders in retired elite athletes (only 0.4% participants had an EAT-26 score above 20). Our results suggested that the athlete’s nutritional behavior during the sport career does not induce mid- and long-term eating disorders. During their career, athletes only control their body mass in order to avoid gaining fat mass before competitions without developing an eating disorder [36,40]. Cereda et al. [3] described that body mass gain observed in obese participants may be due to a hyperphagic state following the diet whereas athletes appeared to maintain food intake control during their career as well as after retirement [41].

The main limitation of the present study could be that data were provided by phone interviews and some of them focused on past event. The comparison between self-reported and direct measurement of body weight supported the accuracy of the data we obtained from interviews. Moreover, concerning retrospective data, to our knowledge no adequate data base was previously built in French elite athletes population, inducing that no other method could be adopted to observe long-term effects of weight cycling.

## Conclusion

The present study shows that there is no particular effect of weight cycling on post-career BMI of retired elite athletes, independently of diets undertaken during their career. Similar patterns of BMI changes were observed in retired athletes and in the general population. The high level of physical activity of retired athletes could explain this lack of repeated diets effects on body mass evolution after retirement.

## Perspectives

While our study did not contain a follow-up of body composition, it might be interesting to link both data (BMI and body composition) in order to evaluate the evolution of bodily compartments with the number of diets. Such a follow-up could allow for an assessment of the actual pressure on body weight management and its effect on eating behaviors as well as body composition changes after retirement.

## Abbreviations

BMI: Body mass index; EAT-26: Eating attitude test

## Competing interests

The authors declare that they have no competing interest.

## Authors’ contributions

LAM has made substantial contributions to conception, design, acquisition of data, analysis and interpretation of data and has been involved in drafting the manuscript or revising it critically for important intellectual content. MB has made substantial contributions to conception, design, acquisition and analysis of data. MT has made substantial contributions to analysis and interpretation of data. HN has been involved in drafting the manuscript or revising it critically for important intellectual content. RM has made substantial contributions to acquisition and interpretation of data. SB has made substantial contributions to acquisition and interpretation of data. JFT has made substantial contributions to conception, design, interpretation of data and has been involved in drafting the manuscript or revising it critically for important intellectual content. FD has made substantial contributions to conception, design, analysis and interpretation of data and has been involved in drafting the manuscript or revising it critically for important intellectual content. All authors have given final approval of the version to be published.

## Pre-publication history

The pre-publication history for this paper can be accessed here:

http://www.biomedcentral.com/1471-2458/13/510/prepub
